# Ultra-Morphology and Mechanical Function of the Trichoideum Sensillum in *Nabis rugosus* (Linnaeus, 1758) (Insecta: Heteroptera: Cimicomorpha)

**DOI:** 10.3390/insects13090799

**Published:** 2022-09-01

**Authors:** Shashikanth Chakilam, Jolanta Brożek, Łukasz Chajec, Izabela Poprawa, Rimvydas Gaidys

**Affiliations:** 1 Faculty of Mechanical Engineering and Design, Kaunas University of Technology, 51424 Kaunas, Lithuania; 2 Faculty of Natural Sciences, Institute of Biology, Biotechnology and Environmental Protection, The University of Silesia in Katowice, Bankowa 9, 40-007 Katowice, Poland

**Keywords:** *N. rugosus*, trichoideum sensillum, mechanoreceptor, morphology, scanning electron microscopy, transmission electron microscopy, 3D model

## Abstract

**Simple Summary:**

*Nabis rugosus* is a representative of the Nabidae family belonging to the heteropteran group. It is a predator of tiny insects and has different sensory receptors that detect environmental changes. The present study focuses on the antennal sensilla of *N. rugosus*, mainly on the trichoideum sensillum as mechanoreceptors for detecting various tactile factors surrounding the insect. The morphology of trichoideum mechanosensillum in *N. rugosus* was modelled as a three-dimensional structure from the derived data sets using SEM and TEM. Specific inner features of the sensillum are also presented, which will be useful to build a biosensor for detecting physical environmental parameters.

**Abstract:**

The present study aims to investigate the morphological features of the antennal sensilla by using SEM and TEM. The construction of a 3D model of trichoideum sensillum using Amira software is presented in this paper. Five sensillum types, namely trichoideum, chaeticum, campaniformium, coeloconicum, and basiconicum, were recorded. This model exhibits the mechanosensillum components, including the embedded hair in a socket attached by the joint membrane and the dendrite connected to the hair base passing through the cuticle layers. TEM images present the dendrite way, micro-tubules inside the dendritic sheath, and terminal structure of the tubular dendrite body and so-called companion cells included in the receptor, e.g., tormogen and trichogen. The parameters noted for the external structure and ultrastructure of the mechano-receptor indicate that they are specific to a particular type of sensillum and would be useful in developing the model for a biosensor. Results show that bio-inspired sensors can be developed based on morphological and ultrastructural studies and to conduct mechanical studies on their components.

## 1. Introduction

Damsel bug, *Nabis rugosus* belongs to the Nabidae family (Heteroptera: Cimicomorpha). Nabidae in the older classification consisted of four subfamilies: Nabinae, Prostemminae, Velocipedinae, and Medocostinae [1,2]. The last classification of Nabidae includes only two subfamilies, Nabinae and Prostemminae [3]; the other two have been recognized as a separate families. Species of the Nabidae family play a crucial role in preserving environmental and ecological stability among the numerous predators hunting other small insects [3,4]. The sensory organs of insects, especially sensilla, are essential in perceiving the ecological changes around them. Mechanosensation through direct touch can also be involved in predator–prey interactions. Ciliated mechanosensory cells that likely mediate such behaviours have been described on the body surface of a large diversity of aquatic invertebrates [3]. These cells are tuned to different types of mechanical stimuli and can mediate prey location and approach, prey capture, or defensive startle and escape responses [5]. Mechanosensitivity in cilia has been reviewed by Wiederhold [6]. Nabidae species have a wide variety of sensilla on the body that detects chemical and mechanical stimulus in the environment [7]. Many researchers have studied the morphology of these sensilla, especially the antennae in different taxa of Heteroptera; however, the antennal mechanosensilla in Nabidae are poorly known [8,9].

Sensilla that respond to mechanical stimuli are called mechanoreceptors [10]. These are scattered throughout the surface of the insect’s body. Tactile, wind currents, vibrations, and gravity are mechanical stimuli caused by environmental forces or by the body’s internal forces [11]. Mechanoreceptors embedded in the insect’s cuticle absorb biomechanical stresses and external stimuli. The trichoid sensilla are the most superficial and highly abundant mechanoreceptors [12].

Trichoid sensilla are a hair-like structure with different lengths and tapered shapes from the base to the apex of the hair and protruding from flexible sockets. A bipolar neuron is located beneath the hair base [13]. The bipolar neuron consists of a dendrite and an axon. This axon connects to the brain, and the dendrite is partitioned into a dendrite sheath and tubular body (cytoskeletal complex structure) consisting of multiple tiny, tightly packed microtubules [14]. Microtubules in the distal part of the dendrite are uniformly organized inside the tubular body and freely dispersed across the rest of the dendrite [15]. Despite numerous studies of the insect’s sensilla, the ultrastructures of the constituent cells of a mechanoreceptor are sporadically presented in the TEM images. However, in several papers on insects’ different mechanoreceptors, the composition of the tubular body and path of the dendrites was varied [13,15].

Understanding insect morphology has substantially improved with the contributions of electron microscopy (SEM and TEM) compared to light microscopy. Using a beam of electrons has extended the scope of suitable magnification. The surface properties are studied by scanning through an electron beam using a standard SEM [16]. The stack of images obtained from TEM can identify each component of the sensilla, which could be used for a detailed study of the morphology, which helps in understanding complex structures at the cellular level and makes it possible to use them in biomechanics [17].

In this study, the morphology, distribution, and ultrastructure of the trichoid sensilla in *N. rugosus* are investigated using electron microscopes. The study focuses on the trichoideum mechanosensillum’s detailed external and internal structure and its mechanical properties. A combination of mechanical sensitivity and mechanical protection of the dendritic sensors are crucial aspects of tactile hair’s coupling to the exoskeleton and their action, which is necessary for the three-dimensional reconstruction structure of this sensilla with the details of all its components present. This study will help to recognize various types of antennal sensilla in *N. rugosus* and to select trichoideum mechanosensillum to understand the interaction of its different components during mechanoreception. This study of biological sensing mechanisms or mechanoreception of *N. rugosus* helps in the development of a sensitive artificial bio-inspired sensor.

## 2. Materials and Methods

### 2.1. Insect Samples

Specimens of *N. rugosus* were collected from the Silesian Park, Katowice, Poland, and conserved in 70% ethanol for SEM studies, and they were preserved in 2.5% of glutaraldehyde for TEM studies. The species is recognized based on the key of damsel bugs and compared with dry materials in the collection of the Zoology Research Team, the University of Silesia in Katowice (DZUS).

### 2.2. Preparation of Samples for Scanning Electron Microscopy

From ethanol materials of *N. rugosus*, the head was detached along with its antennae using scalpels under Olympus stereomicroscope (SZX7) and then shaken in 70% ethanol (1 min) using an ultrasound cleaner (Polsonic, Warsaw, Poland) to remove any dirt particles. Dehydration of the whole antennae was performed with a series of ethanol (70%, 80%, and 90%) for 15 min and bathed twice with 100% ethanol for about 10 min before drying at room temperature. The materials prepared for observation by SEM were kept on carbon tapes and subjected to a 20 nm layer of gold spray sputter coating in the sputter (Quorum 150T ES plus—Quorum Technologies, Laughton, East Sussex, UK) to improve the conductivity of the sample surface. Later, the gold-coated carbon-taped specimens were transferred into the closed chamber of SEM, and images were captured with Phenom XL scanning electron microscope (Phenom-World, Eindhoven, The Netherlands) and Hitachi UHR FE-SEM SU 8010 (High Technologies, Tokyo, Japan) at the Faculty of Natural Sciences, the University of Silesia in Katowice, scanning microscopy laboratory [18,19].

### 2.3. Preparation of Samples for Light and Transmission Electron Microscopy

The antennae of *N. rugosus* were cut from the head; the pedicel was divided into two small pieces and then fixed in 2.5% glutaraldehyde prepared in 0.1 M sodium phosphate buffer (pH 7.4, 4 °C, 24 h). After fixation in glutaraldehyde, the material was washed in phosphate buffer (3 × 30 min, at room temperature (RT)), postfixed in 2% osmium tetroxide (2 h at RT), then washed three times by phosphate buffer for 10 min at RT. The samples were dehydrated. Again, the material was dehydrated in the series of ethanol (30, 50, 70, 90, 96, and 100% for 10 min, 10 min, 15 min. 15 min, 15 min, and 4 × 15 min, respectively at RT), a mixture of 100% ethanol and acetone (1:1, 15 min), acetone (2 × 15 min), incubated in a solution of acetone and epoxy resin (1:1, 1.5 h), and then embedded in epoxy resin (Epoxy Embedding Medium Kit, Sigma, Darmstadt, Germany). In boxes with epoxy resin, the pieces of the pedicel were oriented to a longitudinal section. The material was cut into semithin (800 nm) and ultrathin (50 nm) sections on a Leica EM UC7 RT ultramicrotome (Frankfurt, Germany). Semithin sections were stained with 1% methylene blue in 1% borax, analysed, and photographed with the use of Olympus BX60 stereomicroscope and OLYMPUS DP50 camera. Ultrathin sections were mounted on formvar-covered copper grids, stained with uranyl acetate and lead citrate, and analysed using a Hitachi H500 transmission electron microscope at 75 kV. One hundred and fifteen cross images were serially obtained at 50 nm intervals until the dendritic sheath, including the tubular body, was outside the visual field under TEM at an accelerating voltage of 80 kv. All images were taken at a resolution of 1024 × 1024 pixels and saved as TIFF files. Using 96 images of all 115 serial sections obtained in the trichoid sensilla, an effective magnification of ×18 resulted in an effective pixel size of 0.2 × 0.2 μm at the Faculty of Natural Science, TEM laboratory of the University of Silesia in Katowice [19,20,21].

### 2.4. Image Stacking and Surface Generation

The images obtained from the transmission electron microscopy from the sectioned slides of 50 nm were stacked using microscopy image browser software (Electron Microscopy Unit, Institute of Biotechnology, University of Helsinki, Helsinki, Finland). They were oriented, aligned, and cropped; made into an image stack; and saved with the Amira binary model file (*.am). The *.am file was dumped into Amira 6.5 software (Amira 3D2021.2, Thermo Fisher Scientific’s, Waltham, MA, USA), where these microscopic images’ surfaces and volume rendering were generated. The resulting files were edited for colouring and labelling using the graphic editor Adobe Photoshop CS6 and CorelDraw Graphics suite 2021 [20].

### 2.5. Nomenclature and Measuring of Sensilla

Sensilla were identified in accordance with the nomenclature presented in previous studies [10,22,23,24,25,26]. The schema and photos of the ultrastructure’s of the mechanoreceptors in the present studies were evaluated based on the paper of Keil (1997) and other authors [14,27]. The length of the sensilla on the pedicel was measured with the scale bar in the µm in the photos using the linear dimension tool in CorelDraw (Table 1). In addition, an angle between the base of the trichoid (TSa) and chaetic (ChS) mechanosensilla with the pedicel surface was measured using the angular dimension tool in CorelDraw.

**Table 1:** The approximate lengths of the types of sensilla and their arrangement on the antennae are evaluated.

Types, Length, and Arrangement of the Sensilla										
	shorter		longer		longer	shorter				
	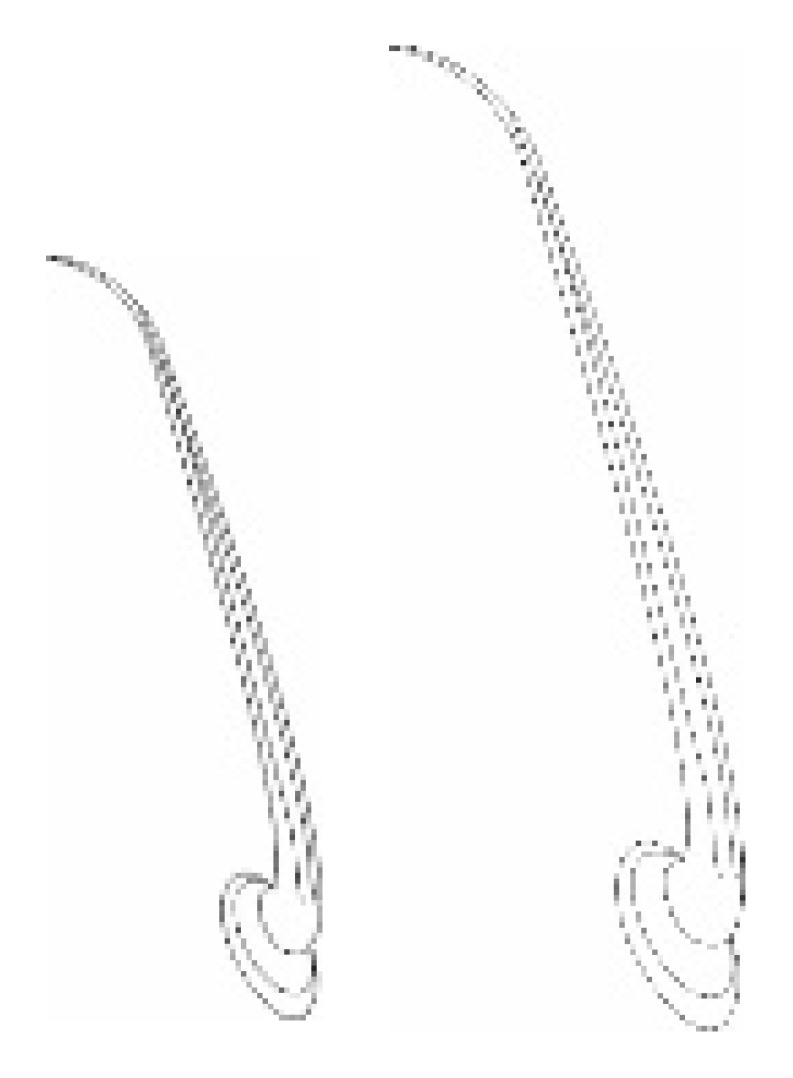		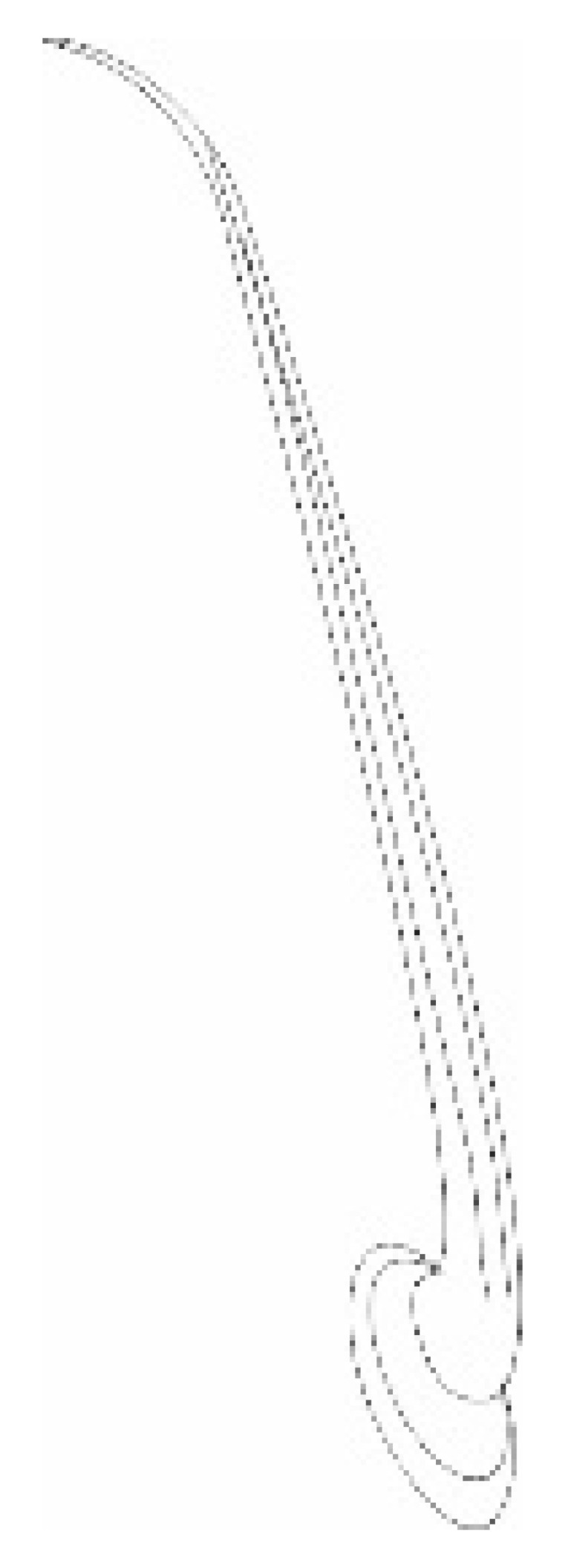	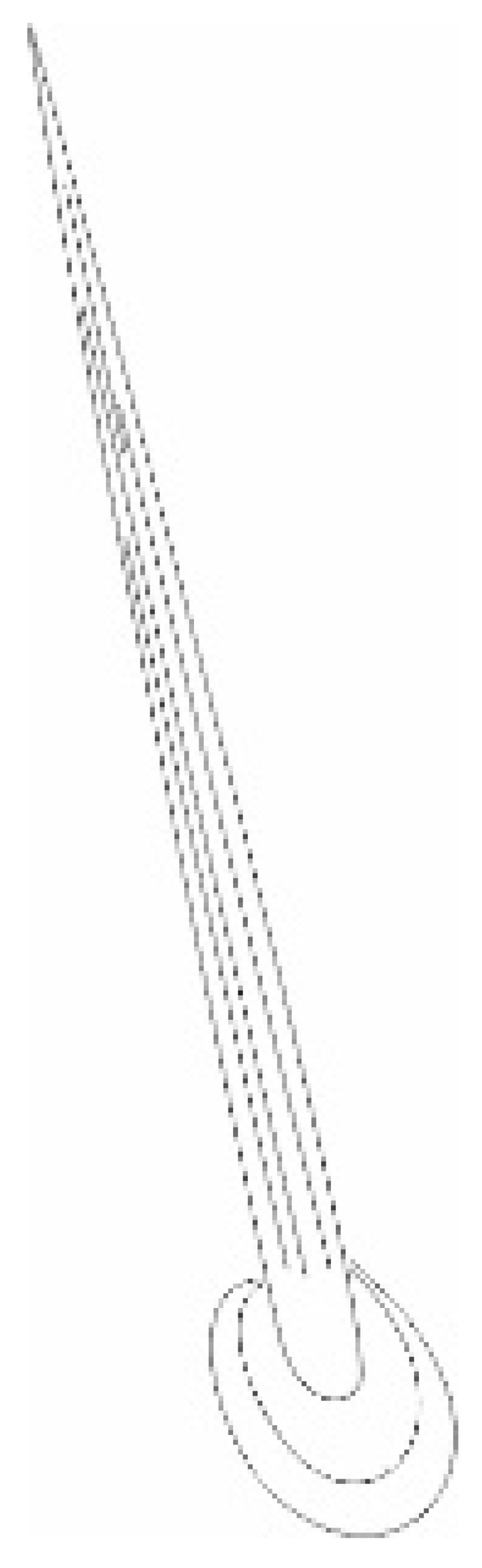	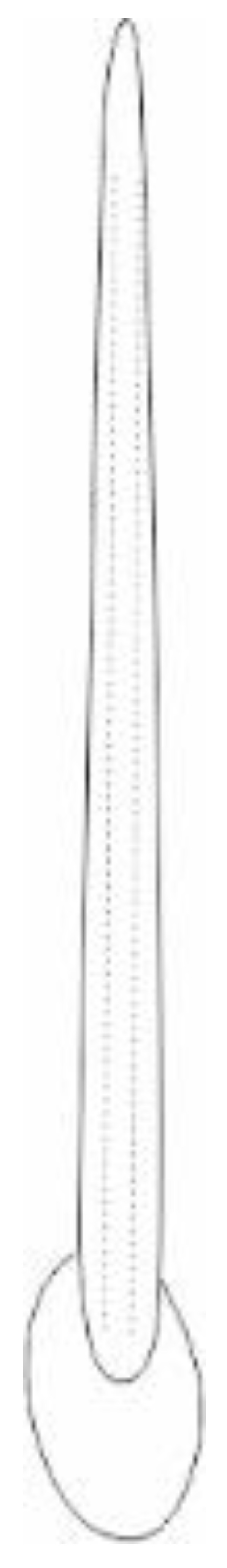	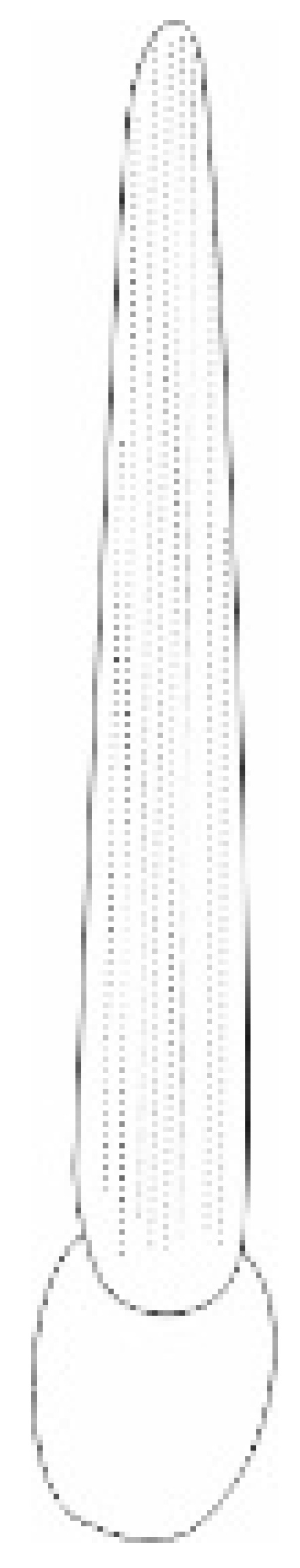	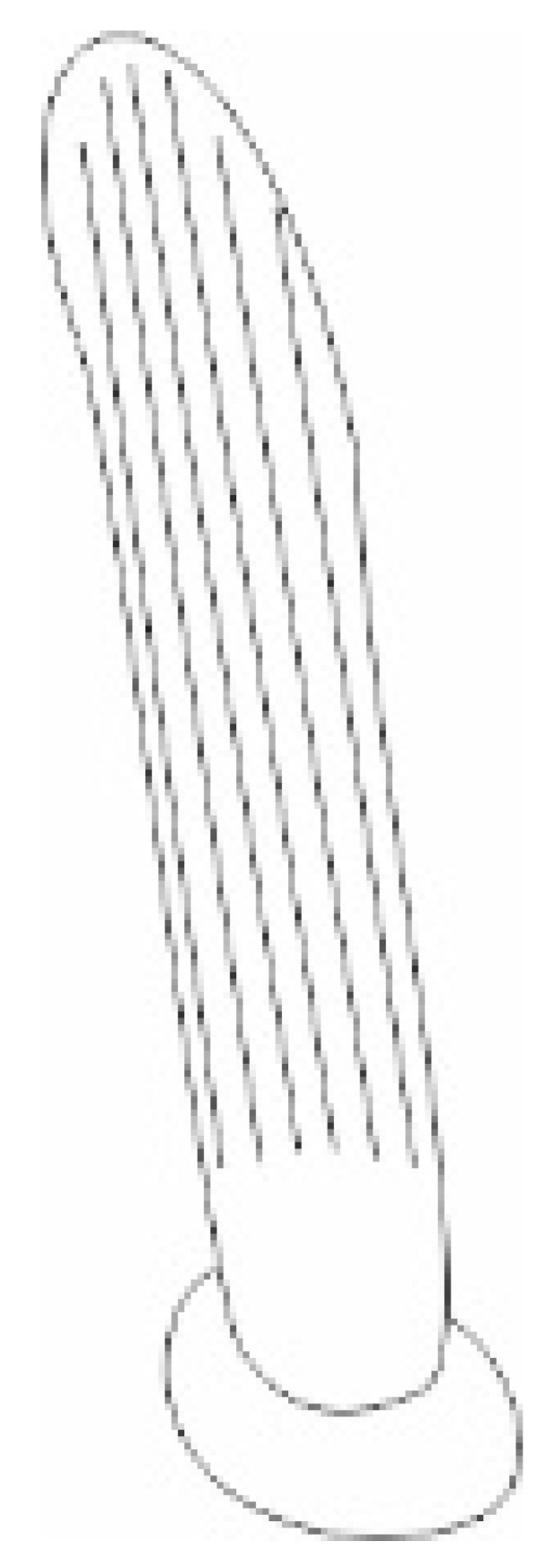	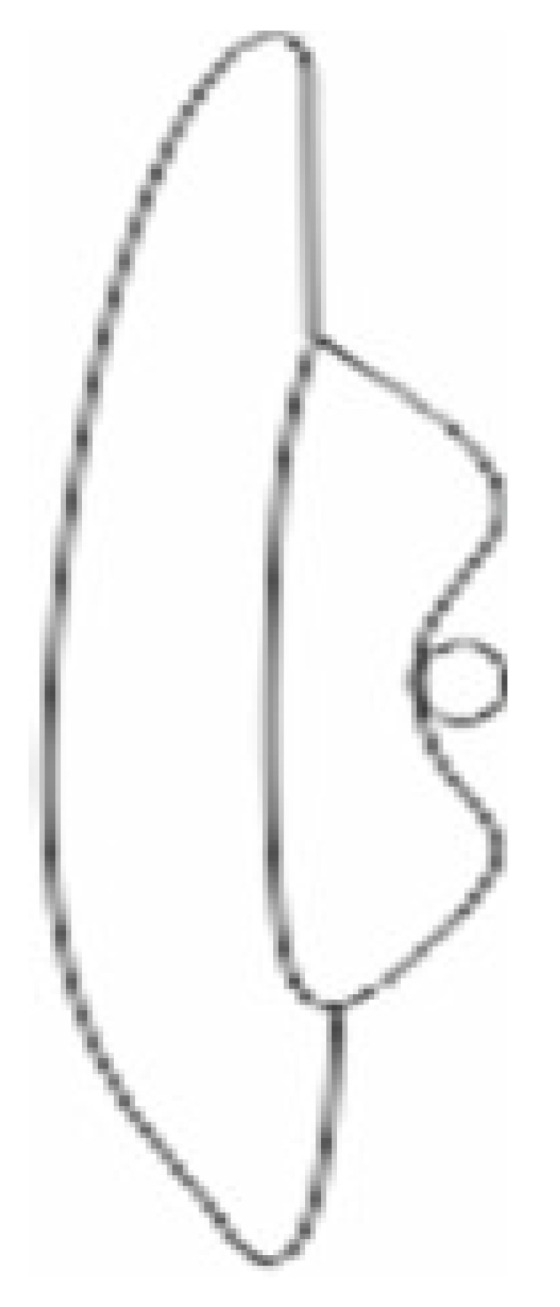	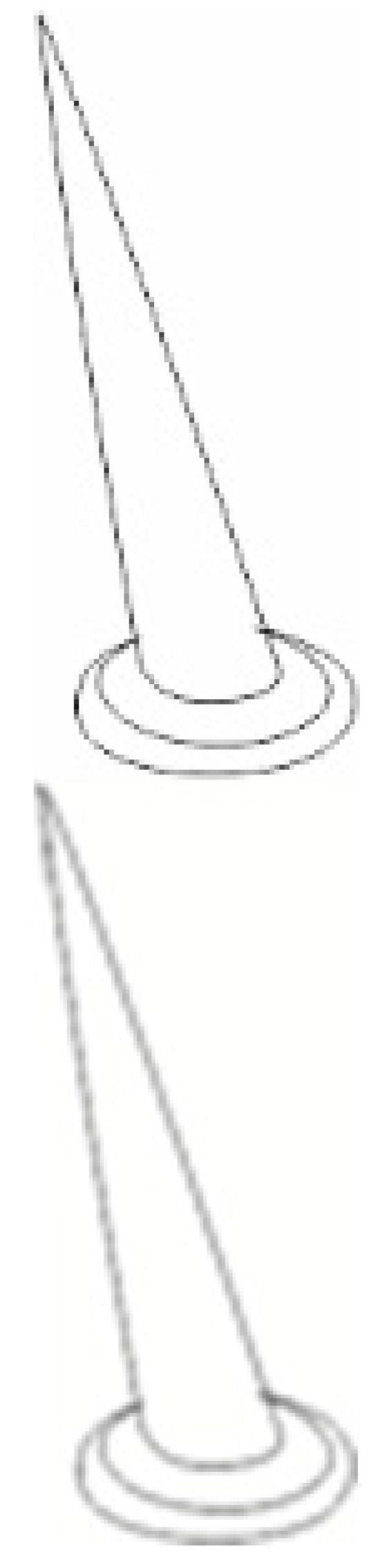	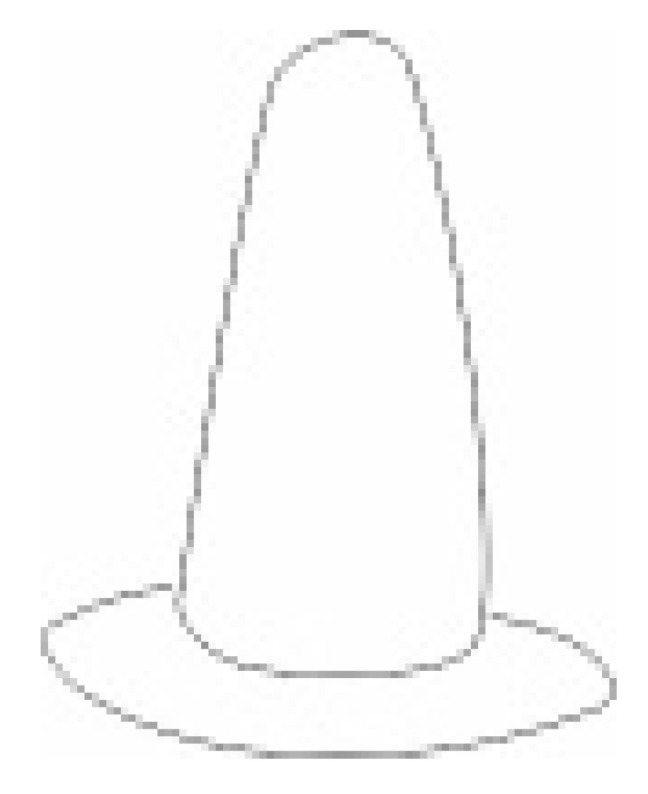
Types sensilla	TSa	TSa	TSa	ChS	TSp1	TSp2	BSp	CS	BSa (pair)	CoS
Length (µm)	22.2 23.5 24.7 27.2 28.0	34.2 36.0 42.2 44.8 45.5	47.6 52.1 58.1 63.7 65.9	53.2 57.8 57.8 62.8 67.9	61.2 65.3 68.5 69.7 66.3	46.2 46.7 46.3 45.6 47.6	14.4 14.7 11.2 11.0 10.0	8.5 7.9	12.8 12.1	4.6
Scapus (s)	+	+	+	+4	-	-	-	+2	+	-
Pedicel (p)	++	++	+	+4	-	-	-	+2	+	-
Flagellomere (f1)	-	-	+	+11	+++	+++	++8	+3	+	+1
Flagellomere (f2)	-	-	+	++18	+++	+++	+++16	+4	+	+1

**Figure 1:**
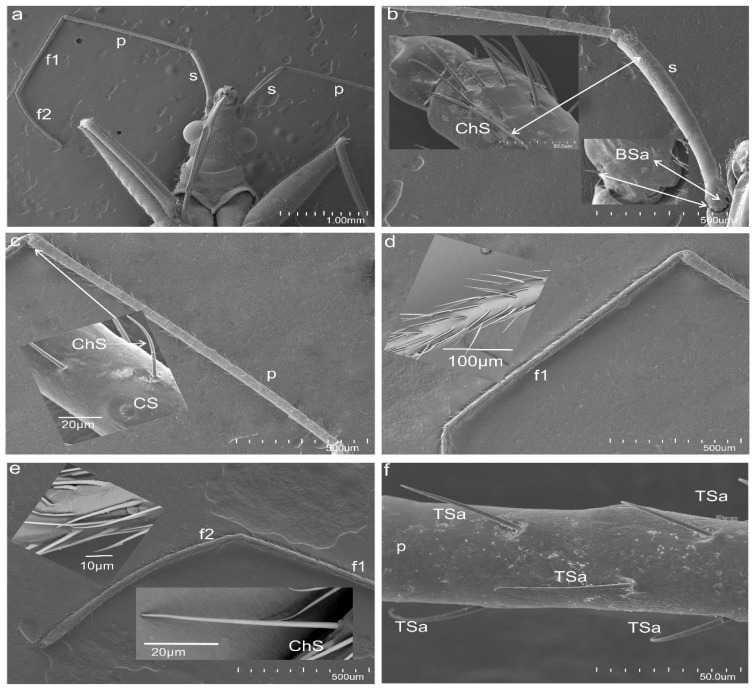
Photograph of *N. rugosus* antennae: (**a**) different antennal segments (scapus (s), pedicel (p), first flagellum (f1), second flagellum (f2)); (**b**) magnification of sensillum types of the scape; chaetic sensillum (ChS) and basiconic sensilla aporous (BSa). (**c**) Magnification of sensillum types of the pedicel (p); chaetic sensillum (ChS) and campaniform sensillum (CS). (**d**) Magnification of the first flagellum (f1). (**e**) Magnification of sensillum types of the second flagellum (f2); chaetic sensillum (ChS). (**f**) Magnification of sensillum types of the proximal part of the pedicel; trichoid sensilla (TSa). Scale bar members of the antenna (**b**–**e**) show the same magnification.

**Figure 2:**
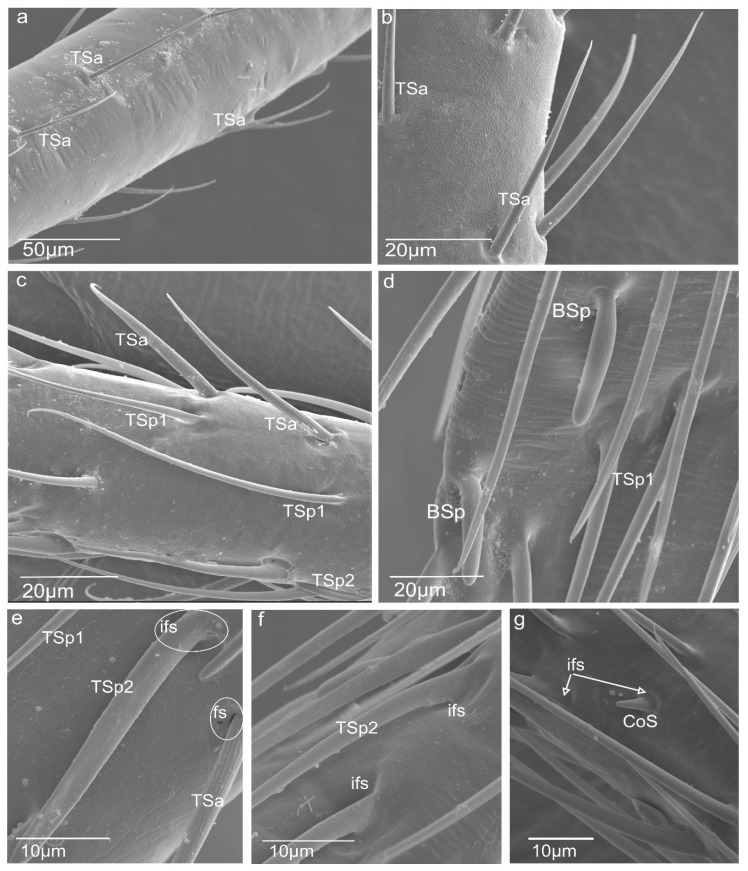
Photograph of *N. rugosus* antennae: (**a**) magnification of the scapus with trichoid sensilla (TSa); (**b**) magnification of the pedicel with trichoid sensilla (TSa) in the distal part; (**c**) magnification of the trichoid sensilla (TSa) and trichoid sensilla (TSp1, TSp2) on the first flagellum; (**d**) magnification of the trichoid sensilla (BSp, TSp1) on the second flagellum; (**e**) magnification of the porous surface of the trichoid sensilla and groove surface of the trichoid a porous sensilla (TSa) with the flexible socket (fs); (**f**) inflexible socket of sensillum (ifs); (**g**) coeloconic sensillum (CoS) observed on two flagellomeres.

+, sporadic (Figure 1b and Figure 2a); ++, some, does not obscure the surface (Figure 1c,f and Figure 2b); +++, numerous, antennae surface densely covered with sensilla (Figure 1d,e and Figure 2c,f), minus, no sensilla, number of sensilla.

## 3. Results

### 3.1. Morphology of N. rugosus Antennae

*N. rugosus* has four-segmented antennae (scapus, pedicel, and flagellum consisting of two flagellomeres (f1, f2)) located on the frontal side of the body, which connects with the head (Figure 1a). Each segment has a different length as follows: 907 µm, 1504 µm, 1204 µm, and 902 µm, respectively (Figure 1b–e).

Distinct morphological types of antennal sensilla based on their shape were identified as trichoid (TS), chaetica (ChS), campaniform (CS), basiconica (BS), and coeloconica (CoS). The external, detailed characteristics indicating the functional types of sensilla are the presence of pores or the lacking (aporous) on the wall sensillum. The critical feature is how the sensilla are embedded concerning the surface of the antennae (in flexible (fs) or inflexible (ifs) sockets), which influences the functional categorization of the sensilla also (mechanosensitive or not). In the study, the socket form was analysed in detail regarding the flexible membrane presence (Section 3.2). The recognized shapes of sensilla, their density, length, and locations are summarized in Table 1. The following is a description of the sensilla types and subtypes:

1. Sensilla trichodea (TS) are hair-like structures. The stem is generally wider in the base and narrow in the apex, which frequently is bent. These sensilla of different lengths are found in larger or fewer numbers on different antennomeres. Their essential character is they are positioned to the surface at an angle (e.g., 25.5–35.1°), laying parallel according to the long axis of the antennae, and slightly raised over their surface.

**Figure 3:**
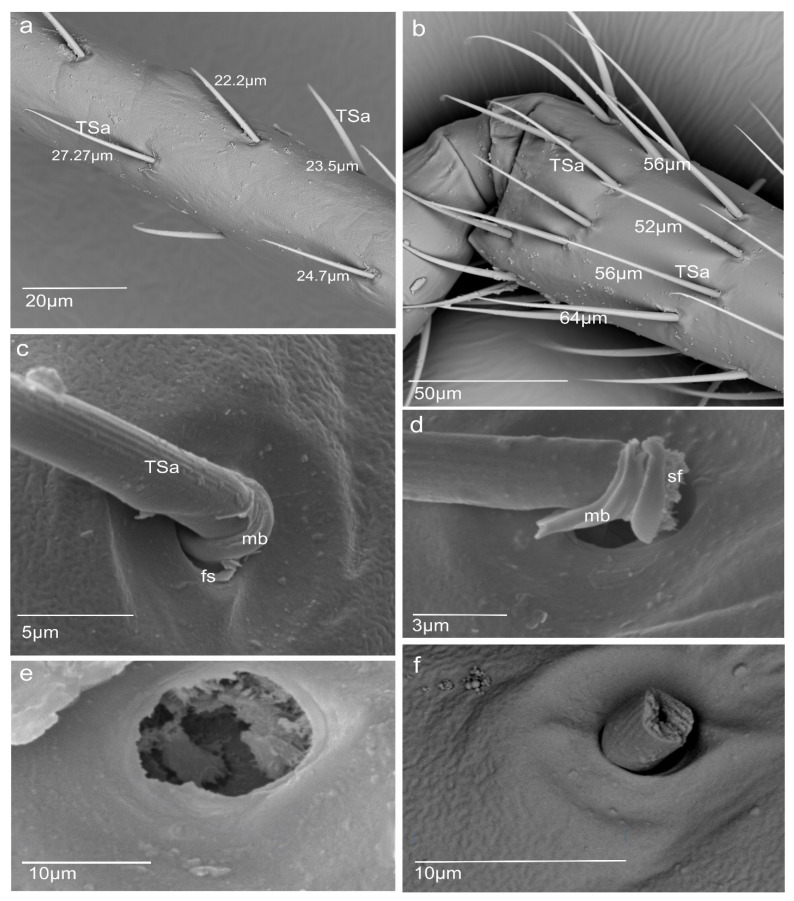
Different length of Sensilla (TSa) and shape of the flexible socket (fs) and (**a**) arrangement of the trichoid sensilla in the proximal and medium part of the pedicel; (**b**) arrangement of the trichoid sensilla in the distal part of the pedicel; (**c**) position of the sensilla embedded in the socket on the surface of the pedicel; (**d**) base part of sensillum with the membrane after removing it from the socket, when suspension fibre (sf) is visible; (**e**) size of the lumen after removing of the sensillum; (**f**) broken sensillum and diameter of the stem of the sensillum near its base.

The trichoideum sensillum subtype (TSa) is straight and hair-like with a slightly ribbed surface without pores (aporous wall sensillum) and are embedded in the flexible socket. The thin membrane at the base of the sensillum is connected to the cuticle, which provides its mobility. The sensilla are of different lengths and various localities on the antennae. On the scapus, in the proximal part, there are short sensilla (L = 28.0; 36.0 µm) and ones longer (L = 52.1; 63.7 µm) in the distal end (Figure 1b and Figure 2a), which are sporadic (+). The trichoid sensilla (TSa) is found also on the pedicel and is shorter in the proximal part (Table 1, see Section 2.5) (Figure 1f, Figure 2b, and Figure 3a,b) but in the distal sensilla is longer (L = 58.1; 65.9 µm) (Figure 2b). On the first and second flagellomeres, longer sensilla (TSa) are singles (Figure 2c), and the short kind do not occur (Table 1).

The trichoideum sensillum (TSp) subtype possesses a characteristic porous wall and inflexible socket (ifs) (Figure 2c–f). Within them are distinguished: longer trichoideum sensillum (TSp1), with length from 60–70 µm and possessing a narrow base of the inflexible socket, and shorter (L = 45 µm) trichoideum sensillum (TSp2) with a wider base of an inflexible socket. These sensilla are numerous on both flagellomeres (f1 and f2) (Figure 2c–f) (Table 1).

2. Sensilla chaetica (ChS) are long (53 to 70 µm), rigid structures that are ribbed and sharpened at the tip and arise from a flexible socket. These sensilla are not numerous, and they were only found as singles in the distal part of the scapus (Figure 1b) and proximal portion of the pedicel (Figure 1b). Still, there are slightly more on the lateral sides of the first and second flagellomeres (Figure 1d,e). The sensilla are positioned on the antennal surface at a larger angle (42°, 48°) than the trichoid sensilla; therefore, they are visible, as they stick out (Figure 1e).
3. Sensilla campaniformia (CS) are oval-shaped structures with a single pore in the middle embedded in the flexible sockets. They are present only a few (2–4) in different places of each of the antennomeres. Their presence is shown on the scapus and pedicel (Figure 1b,c).
4. Sensilla basiconica (BS) represents two subtypes. One of the subtypes (BSa) is short (length approximately 5 µm), smooth, aporous, cone-shaped structures embedded in the flexible socket, occurring in one pair on the edge of the adjacent segments (Figure 1b).

Another subtype of sensilla basiconica (BSp) represents a sturdy, cone-like structure with a grooved porous surface and slightly rounded tip. Their length is about 15 µm. The sensilla are embedded in the inflexible sockets, and the base of the stem is strongly bent. The middle and distal parts of the sensillum lay parallel according to the long axis of the flagellomeres. Several sensilla are singularly arranged on both flagellomeres (f1 possesses about 8 such sensilla, whereas f2 has 18) (Figure 2d).

5. Sensilla coeloconica (CoS) are peg-like and short, with smooth surfaces embedded in a shallow cavity of cuticles in inflexible sockets. Only one such sensillum was observed on each flagellomere (Figure 2g).

### 3.2. A Detailed Description of the Trichoideum sensillum on the Pedicel with the Structure of the Socket

**Figure 4:**
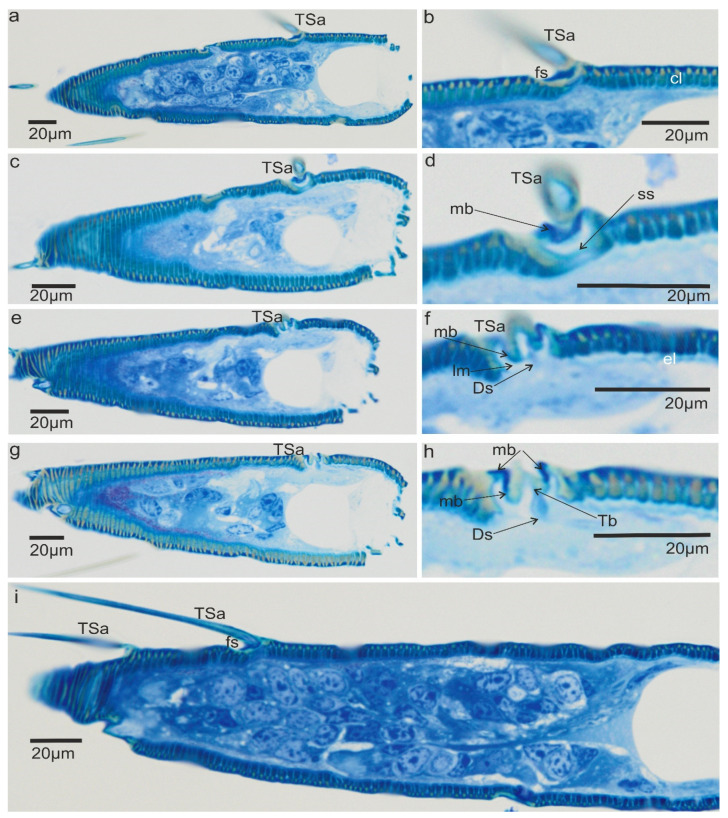
Microscopic photograph of the ultra-section of the flexible socket and termination of the tubular body of trichoideum sensillum in *N. rugosus*, longitudinal section by part of the pedicel: (**a**,**b**) trichoideum sensillum visible from the cuticular side; (**c**,**d**) further longitudinal section, with the shape of the socket and membrane visible in the deeper layer of the cuticle; (**e**,**f**) inner side of the socket and lymph cavity (lm) and part of the dendrite of the mechanoreceptor; (**g**,**h**) dendritic way (Ds) and tubular body terminated at the base of the trichoideum sensillum; (**i**) magnification of the other trichoid sensilla and the base of the sensillum in the socket. Cl, cuticular layer; f, flexible socket (fs) in trichoideum sensillum (TSa); el, epidermal layer; mb, socket membrane; ss, socket septum.

The trichoideum sensillum (TSa) is found on all sides of the pedicel and is rarely arranged. It is easy to identify because of its outstanding regular arrangement in the proximal and middle of the pedicel and similar length in the range from 22 µm to 45 µm (N = 8) (Table 1) and steep insertion angle in the range from 25.5° to 32.8°. The end of the sensillum is usually bent and flexible, which is observed in the many hairs. The diameter of the sensillum at the tip is 0.55 µm. The proximal end of the sensillum shaft is connected to the socket by the socket membrane (mb) (Figure 3c,b), which protrudes distally (towards the base of this sensillum). Figure 3e,f shows the shape and size (W: 31.99 mm (= 11.3 µm) L: 41.46mm (=14.6 µm)) of the socket after removal of the sensillum and the diameter of the stem of the sensillum (W: 3.83 mm (=2.97 µm)) near its base. According to the structural appearance of the socket membrane (mb), the same kind of material also lines the inner surface of the socket (Figure 4a–h). In longitudinal sections, the position and size of the flexible socket and its structural components ((socket septum (ss), socket membrane (mb)), and the inner structures of the pedicel (the thickness of the cuticular layer (cl) and epidermal layer (el)) (Figure 4a–i) are visible. The semi-thin slides (Figure 4e–h) revealed the fine structure of the basal region of the trichoideum sensillum, including the dendrite attachment area (Ds) and the probable area of the tubular body (Tb). The trichoideum sensillum (TSa), which is distributed on the pedicel, was selected to study the internal structural organization of its components and morphology (Figure 1c and Figure 2a). Other types of sensilla, such as chemosensilla and thermo-hygrosensilla in this segment, are not observed in SEM; therefore, this antennal area for the study of the flexible trichoid mechanosensillum was selected in this study.

### 3.3. Ultrastructures of Trichoid Mechanosensillum Using TEM

**Figure 5:**
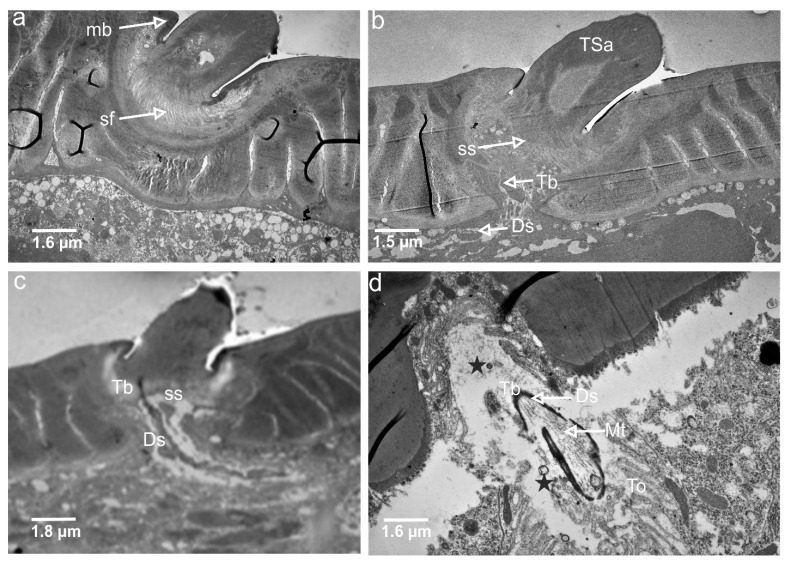
TEM photograph explores the ultrastructure of a longitudinal/oblique section of the flexible socket and section of the tubular body of trichoideum sensillum of *N. rugosus* antennae: (**a**) section by flexible socket, where the shape of the membrane and suspension fibre is visible; (**b**,**c**) deeper section of the mechanoreceptor, where the dendrite coupling to the base of the socket is clearly visible; (**d**) tubular body in the oblique section. ss, socket septum; Ds, dendrite sheath; sf, suspension fibre; Mt, microtubules; To, tormogen cell; black stars, lymph cavity; mb, socket membrane or joint membrane; Tb, Tubular body; TSa, trichoideum sensillum.

In the longitudinal section (Figure 5a,b), the socket membrane (mb), suspension fibre (sf), and socket septum (ss) are documented in the ultrathin slides.

**Figure 6:**
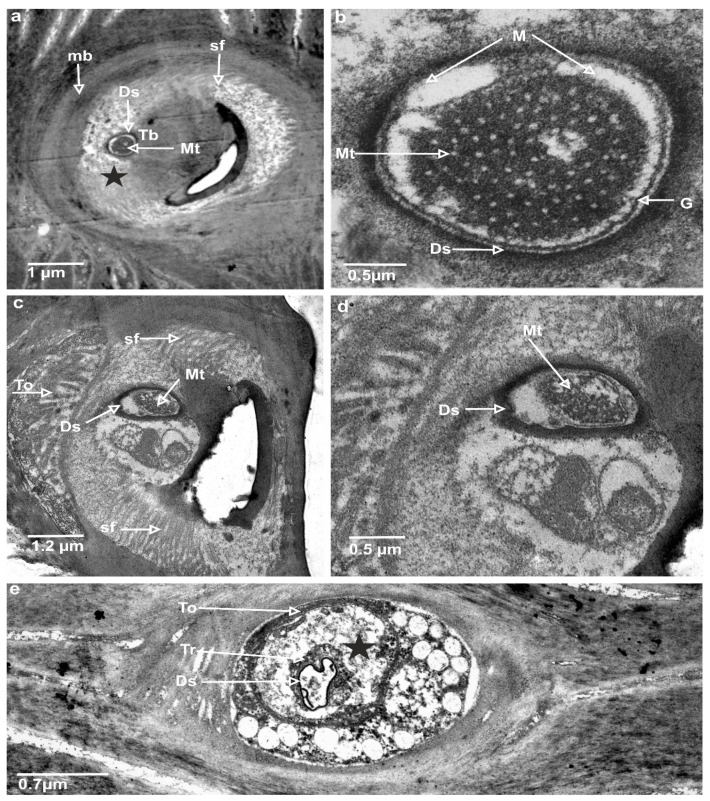
TEM photograph explores the ultrastructures of the trichoid mechanoreceptor: (**a**) cross-section at the base of trichoid mechanosensillum, where tubular body (Tb) is visible; socket membrane or joint membrane (mb) (**b**) magnification microtubules (Mt) of the tubular body; (**c**) profound location of the mechanoreceptor, where tubular body is wider in this section of trichoid mechanosensillum; (**d**) enlargement and the shape of the tubular body; (**e**) tormogen cell (To) with the sensillum lymph cavity (marked star) and trichogen cell (Tr), dendrite sheath (Ds), line of granules (G), suspension fibre (sf), and membrane (M).

**Figure 7:**
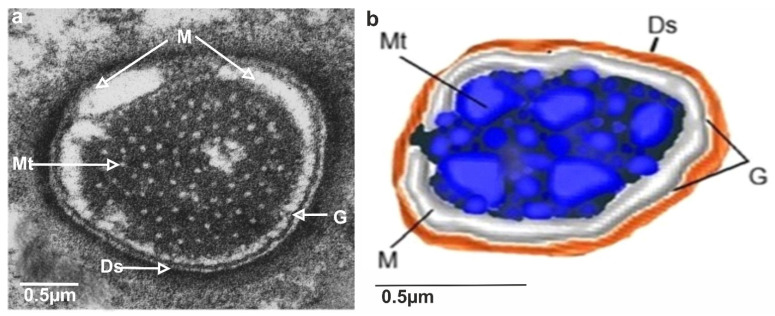
Model of the tubular body of trichoideum mechanosensillum in cross-section in *N. rugosus*: (**a**) original TEM photo of cross-section; (**b**) compilation of numerous thin slides to analyse particular components. Ds, dendrite sheath; G, line of granules; sf, suspension fibre; Mt, microtubules; M, membrane.

**Figure 8:**
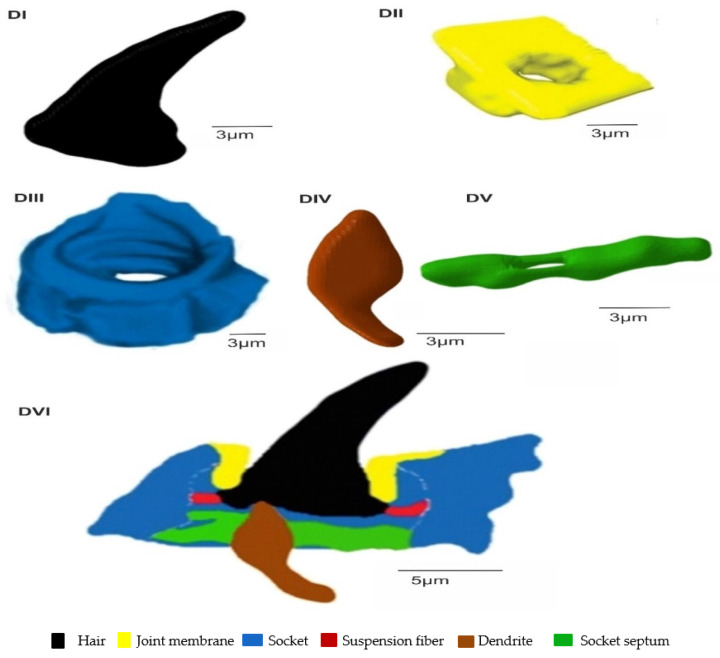
Three-dimensional model of trichoideum sensillum’s internal organizational structure, developed by Amira software. Individual components are (**DI**) hair; (**DII**) joint membrane; (**DIII**) socket; (**DIV**) dendrite; (**DV**) socket septum. (**DVI**) Sectional view model of the sensillum.

The TSa hair base and socket are connected with suspension fibres that control the hair base’s extensive movement. There is one unbranched dendrite ending at the trichoid mechanosensillum base (Figure 5c). It is surrounded by a dendrite sheath (Ds) (Figure 5c,d), which is also presented in the oblique section via dendrite (Figure 5d). As seen in Figure 5d, the dendrite sheath involves numerous individual microtubules (Mt). The tubular body is primarily positioned at the terminal section of the dendrite sheath and is tightly packed with microtubules. The dendrite sheath localized to the hair base is supported by the socket septum (Figure 5c). External of the dendrite sheath, the tormogen cell (To) with microvilli increases in diameter near the socket (Figure 6c). The rounded dendrite sheath lies within the distal part near the base of the hair (Figure 6a). By large magnification (Figure 6b) of a cross-section of the tubular body, we observe that the space between the densely packed microtubules (white dots) is filled with moderately electron-dense material (dark area). The diameter of the tubular body is about 150 nm, and microtubules (Mt) in *N. rugosus* that are around ninety in number and well-visible in the cross-section of Figure 6b. The space between the dendrite sheath and the tubular bodies is filled with granules (G), which contact both the dendrite sheath (Ds) and the dendritic membrane (M) (Figure 6a,b). Below the tip, in the lower part, the dendrite sheath is more oval-shaped and flat (Figure 6c,d), and microtubules are not tightly packed. The dendrite passes through the subcuticular region, a sensillum lymph cavity (lm) formed by concentrically stacked trichogen (Tr) and tormogen (To) cells, with microvilli up to the hair base (Figure 6e). There is no observed direct coupling between the dendrite sheath and the hair base. However, it is seen that the end of the dendrite sheath with the tubular body almost touches the base of the hair on one side (Figure 5c). The extracellular material surrounding the dendrite sheath in the proximal part also forms the socket septum. The very thin layer of the extracellular material will probably joint the end of the dendrite with the tubular body to the base of the hair. The cross-sectional dendrite with the tubular body near the base of hair and a structure model developed by compilation of numerous thin slides are presented (Figure 7a,b). Different components of sensilla (hair, dendrite, joint membrane, socket, socket septum) and sectional view of TSa after reconstruction of the 3D model are shown (Figure 8DI–DVI).

## 4. Discussion

### 4.1. Type of the Antennal Sensilla in N. rugosus

In *N. rugosus*, five essential types of sensilla (trichoid, chaetic, campaniform, coeloconic, and basiconic) were identified morphologically by shapes, kind of sockets, and porous or aporous wall of the sensillum. These findings agreed with various previous studied conducted on Hemiptera [7,28,29,30,31,32]. However, in some species of heteropterans (*Leptoglossus zonatus* (Dallas), the placoid sensilla were additionally distinguished [33]. In several phytophagous species [29,31,32] were recognized the following sensilla: trichoid sensilla, type 1 and 2 (ST1 and ST2), long and short basiconic sensilla (SB1, SB2, and SB3), knob-shaped basiconic sensilla, long chaetic sensilla (Sch), and coeloconic sensilla (Sco). The current morphological data were used to divide sensilla into the functional categories of chemoreceptors, thermo-hygroreceptors, and mechanoreceptors according to essential papers by Slifer [22] and Alther and Prillinger [34]. Usually, sensilla with a porous surface are chemoreceptive (olfactory) sensilla in contrast to aporous mechano—and thermo—hygrosensilla [34] except for the contact-chemoreceptive sensilla, which are bimodal (taste–touch) and possess a terminal pore and flexible socket [35]. Based on the morphology of the trichoid sensilla (TS), in *N. rugosus* was distinguished a tactile trichoid mechnosensilla (TSa) with aporous wall and flexible socket (fs) and olfactory trichoid chemosensilla (TSp) with porous wall and inflexible socket. Generally, both types of sensilla are popular in insects [25,35]. This trichoid mechnosensilla (TSa) are different in length (short and long) and were found singularly on the flagellomeres; however, on the scapus and pedicel, they are dominant. The short are more numerous on the pedicel than on the scapus. The longer is grouped distally on each. Similarly, two types of trichoid mechanosensilla were documented in *Odontopus nigricornis* (Stall) and *N. viridula* L. (Heteroptera: Pentatomomorpha) as long, thin hairs and slightly shorter hairs with flexible sockets at the bases [7]. Aporous, striated trichoid sensilla were also identified in *L. lineolaris*, which were numerous on the pedicel [29]. The mechanoreceptive function of trichoid sensilla was also pointed out in other terrestrial heteropterans such as in *Oncopeltus fasciatus* (Dallas) (Lygaeidae), *Lygaeus kalmii* Stål (Lygaeidae), and *N. parvus* (Westwood) (Alydidae) [36,37,38], respectively. The mechanoreceptive role is also attributed to the chaetic sensilla, which is present *N. rugosus*. The stiff and long chaetic mechanosensilla (ChS) is not numerous and sporadically localized laterally in the distal part of the pedicel and on both flagellomeres (f1 and f2). The base and shaft of the chaetic sensillum are more extensive than in the trichoid mechanosensillum (TSa). In *O. nigricornis* and other species, at the periphery of the antennae, chaetic sensilla with bulbous bases were also observed [7]. Long sensilla chaetica are adapted for the reception of tactile stimuli, air currents, substrate vibrations, and shocks registered by the sensilla from exploratory movements of the antennae [39].

Another type of mechanosensilla followed on the antennae represents a proprioceptive basiconic sensillum (BSa) positioned on the edge of the adjacent segments. The aporous sensilla basiconica (BSa) embedded in the flexible sockets is associated with position control and occupies a stable position in a bending place between particular segments. In some species, e.g., *L. zonatus*, the proprioceptive sensilla are described as small, smooth trichoid sensilla found precisely on the joints between the pedicel and scape [33]. Sensilla are arranged conservatively in the places of bending in insects [23,35]. According to McIver [23] and Zacharuk [40], campaniform sensilla are mechanoreceptors commonly present in insects and are situated in areas of the cuticle that are subject to stress. These sensilla are found on different parts of the antennae and have usually been reported on the scape, near the segmental joints, or the membrane on the top of the pedicel [27]. In *N. rugosus*, the several disc-shaped campaniform sensilla are irregularly spread on each antennal segment.

Many researchers have examined and confirmed that the mechanosensilla are in different shapes and sizes and have different distribution patterns over the antennae. However, the development and functionality of the mechanosensilla are still similar, which respond to any mechanical stresses in the cuticle part [23,35,41]. A relatively higher abundance of trichoid mechanoreceptors (TSa) and not numerous chaetic sensilla (ChS), campaniform sensilla (CS), and basiconic sensilla aporous (BSa) could be considered as potential detectors for various mechanical functions.

The presence of multiporous basiconic chemosensilla (BSp) and trichoid chemosensilla (TSp1 and TSp2) reflects the ability of the antennae to perceive chemical stimuli. Olfactory sensilla are characterized by a porous cuticle and inflexible socket allowing the entry of different odour molecules [24,42,43]. According to the present study, olfactory function in *N. rugosus* is conducted by three subtypes of sensilla (TSp1, Tsp2, and BSp). The porous trichoid chemosensilla (TSp1 and Tsp2) probably represents a single-walled pore sensilla type described by Slifer [22].

This type of trichoid sensilla is involved in chemoreception in the present studied species and, as shown in many other species of Heteroptera (*L. lineolaris*, *O. nigricornis* (Stål) *N. viridula* (L), *N. parvus* (Westwood)), the flagellomeres are equipped with long sensilla with single-wall pores, suggesting their olfactory role [7,38,44,45]. Depending on the trichoid sensilla’s external and internal construction, they can perform either mechano—or chemo—sensory or both functions [24,40].

The fact that olfactory trichoid sensilla in *N. rugosus* and the species mentioned above were most abundant and different in size on the antenna confirms their importance for broad perception, intraspecific recognition, and pheromone detection [27,29]. The basiconic chemosensilla (BSp) distinguished in *N. rugosus* belongs to the multiporous, grooved, double-walled sensilla. Such types of sensilla play an olfactory role by perceiving long-distance stimuli [22,40]. A similar sensilla type is described as basiconic sensilla (4) in gerromorphan species [26]. In three stink bug species (Pentatomidae), the putative olfactory function was indicated for sensilla ST1, SB1, and SB2 in detecting male-produced sex pheromones and odours derived from the host plants. Moreover, differences were detected in the abundance and arrangement of these sensilla over the antennal segments in individuals of the same species and among the species studied [31]. Like in *N. rugosus*, the most significant number of olfactory sensilla were found on the flagellomeres in *L. zonatus* [33], *N. parvus* (Westwood), and *Rhodnius prolixus* (Stal). Such arrangement and accumulation of these types of sensilla of both flagellomeres could be an adaptation to improve olfaction.

The thermo-hygroreception is common and well-recognized in terrestrial and water insects [8,26,34]. Mainly, the function is connected with the unique structure of the ampulacea and coeloconic sensilla [24,26]. In *N. rugosus*, responsible for thermo-hygroreception are two coeloconic sensilla (CoS), singularly localized on each flagellum. Generally, these are non-porous, peg-shaped sensilla inserted in shallow cavities. Such sensilla are usually present in fewer numbers or singly [34,35], which was confirmed in the present study.

### 4.2. Structural Components of the Mechanoreceptor Sensilla

The ultrastructure of the insect’s mechanoreceptor was detailed and indicated in the six types of mechanosensilla (bristles, trichobothria, filiform, campaniform sensilla, and scolopidia) by Keil [14] and one type of the short trichoideum sensillum in Brown planthopper, *Nilaparvata lugens* (Fulgoromorpha: Delphacidae) [15]. The general cell organisation of the mechanoreceptors in different types of sensilla are almost identical [14], but the different number of the microtubules is significant for the action of the sensillum.

In a distal tip of the dendrite, there is a highly ordered cytoskeletal complex, the “tubular body”, that is specific for the mechanoreceptor in which, in different insects, mechanosensilla are formed by a few dozen up to about 1000 relatively short (1–2 µm) microtubules. The microtubules are tightly packed and connected to the dendritic plasma membrane via numerous short and stout connectors [46].

External morphology and general classification of the sensilla [23,24] indicated that the outer flexible trichoideum sensillum stem differs from the bristle’s stiff stem or chaetic sensillum stem. External sensillum hair comprises cuticular layers such as exocuticle and endocuticle. Thus far, the ultrastructure of the tubular body in the flexible trichoideum sensillum has not been examined on the antennae in the heteropteran species. Antennae are equipped with different trichoid sensilla sizes, as shown in Figure 1c–f and Figure 2a–g.

In the present research of *N. rugosus*, it is observed that the longitudinal section has a clearly visible, large sensillum lymph cavity of the tormogen cell (Figure 3c,d). The dendrite path from the epidermis through the endo and exocuticle up to the base of the trichoid hair and all socket elements are clearly observed. The large tormogen cell seems to be more active in transport and secretion in mechanoreceptors, and the same holds true for the trichogen cell in the olfactory sensilla [27].

The inner structures of dendrite near the base of the hair revealed the presence of a tubular body (Tb). Figure 6a and Figure 7a,b show the dendritic sheath (Ds) connected with the plasmic membrane (M) through-line of the granules (G), which corresponds to the attachment filaments between the plasmatic membrane and dendritic sheath, according to Keil [14]. This dendritic sheath (Ds) surrounds the structured filaments at its distal end, known as the tubular body. This tubular body is tightly packed with many microtubules, which are very small and are integrated with the membrane (M) by tiny rigid connectors named membrane-integrated cones (MMCs), pointed out by Keil [14], but invisible presently in Figure 6a. These MMCs are found in the area where the mechanical forces directly occur. A similar structure is analysed in other species e.g., *N. lugens* [15]. The trichoideum sensillum’s position and form on the antennae in *Nabis* have confirmed the mechanical function. Compared to the other mechanosensilla, the number of the microtubules in *N. rugosus* (about 90) is approximately the same as campaniform sensilla on the base of the haltere of *Drosophila* (about 100) [47] but more significant in number than that of *N. lugens* (about fifty) in sensillum located ventrally on the abdomen [47]. Furthermore, in other taxa, the number of microtubules in the tubular body varies from 30 in the tsetse fly [48] to approximately 1000 in the cockroach [14].

In insect mechanosensilla, the tubular body was indicated in several studies, but the microtubule number was rarely analysed in detail. The position of the tubular body at the base of the trichoid sensillum seems to be different in varied species and depends on the position of the hair in the socket, e.g., dendrite with an apical tubular body attached in the centre of the base of the seta on the hemelytra of backswimmers, *Notonecta glauca* (Notonectidae: Heteroptera) [49].

In the *N. rugosus*, in the trichoideum sensillum, the tubular body is situated on one side of the hair base, whereas in *N. lugens*, it is in the middle of the hair. The comparison of the sensillum length to the tubular body diameter and the number of different microtubules shows that the length of the sensillum in *N. rugosus* is about 25 µm long and has a long socket axis of about 11.3 µm, which is longer than the sensillum of the *N. lugens*, whose length is 11.8 µm, and the socket axis about 4.8 µm. However, the filiform sensilla on the cerci of crickets are evidently different and significantly longer (between 30–1500 µm in length) in comparison to the above-mentioned species, while their diameter varies from 1.5–9 µm. Their suspension is constructed in a way that allows deflection of each hair in only one exactly defined plane. The filiform sensilla are arranged in a highly stereotyped pattern in rows: within each row, all hairs can be deflected either parallel (longitudinal or L-hairs) or normal (transverse or T-hairs) to the cercus axis. This allows stimulus localization, e.g., of a predator approaching from behind, and triggers the escape response [50]. In *N. rugosus*, on the pedicel, the trichoid mechanosensilla is not numerous and regularly spreads at a sharp angle to the surface, and between each sensillum, there is ample space. This type of arrangement of the sensilla can be explained as the sensilla do not catch one another and are free to react to the mechanical factors of the environment. It seems that is another model of mechanosensitivity besides that on the cerci of crickets.

The socket walls are cuticular and may bear inward-projecting ribs and diaphragms. These projections and the socket’s height and diameter restrict the hair’s movement [23]. These elements are essential in the construction of the bio-mechanosensor.

The diameter of the tubular body is different for each type of mechanosensilla. In the present study on *N. rugosus*, the diameter of the tubular body in the trichoid sensillum is quite large, which is 150 nm compared to 50 nm for *N. lugens* [15].

The internal fine structure of the mechanosensillum of housefly interfacetal hair showed that at the hair base, the dendrite of the neuron terminates in a tubular body only 1.5 mm in diameter, which is filled with about 400 microtubules in systematic order [51]. The latter authors suggested that the short tubular body as well as its eccentric insertion into the hair shaft maybe point to a highly sensitive mechanoreceptor. Based on their single innervation, the mechanosensillum of housefly interfacetal hair could monitor flight speed from the degree of hair deflection caused by wind in general or particular laminar air currents flowing past the eyes during flight. The presented parameters showed that the position and size of the external structures of the sensillum as well as socket and tubular body structures could have significant value in modelling the accurate bio-inspired sensor.

### 4.3. Biological Sensing Mechanism of Trichoid Sensilla

Trichoid mechanosensilla are specialized in sensing the mechanical disturbances due to air, water flow, and vibrations with hair deflection. The sensing of the mechanical impulse involves coupling, transduction, and encoding. The hair laterally protrudes out of the socket, and the hair base is supported by the suspension fibres that restrict the hair’s extensive movements when there is a flow over the hair. The movement of the hair is restricted to a single plane, demonstrating directional sensitivity [47,52]. The mechanical architecture of the hair base and the geometry of the dendrite tip can have a significant effect on receptor sensitivity. In most cases, the dendrite tip and tubular body are flattened in the mechanical impact direction. Some receptors respond to minimal deflection angles and can quickly achieve saturation [46].

The hair shaft of the sensilla works as a first-order lever, where the distal end of the hair tip has large displacement, and slight motion at the basal part will press the dendrite’s tip attached to the hair base [53]. Since the joint membrane is elastic, it helps to convert hair deflection to compressive force onto the dendrite tip. It also prevents the deformation of the hair base and acts as an elastic spring for restoring the hair to its normal position. These stimuli over the dendrite by the hair shaft are known as coupling [54]. This dendrite with dendrite sheath and a tubular body is tightly packed with microtubules, and any pressure applied on the dendrite sheath due to deformation of the hair will generate ion currents, which is considered a transduction process. These induced ion currents, also known as receptor potentials, pass through microtubules and flow into the axon of the central nervous system, which delivers information to the insect brain [14,55]. Schroeder et al. [54] claimed that the joint membrane should be elastic and flexible enough to deflect the hair. The action potential was most substantial when the hair was bent toward the dendrite’s tip connected to the hair base [35,52].

As documented in spiders, the sensitivities of mechanosensory hairs are changeable; in trichobothrium, it is smaller than in other tactile hairs. In the various tactile hairs, in regard to their mechanical properties, the sensitivities are radically different. While being deflected by frictional forces due to the movement of the medium, a trichobothrium’s hair shaft is not bent, with the main reason being the very small elastic restoring moments at its base. These are of the order of 10^−12^ Nm rad^−1^. In the various tactile hairs, in regard to their mechanical properties, the situation is radically different. The elastic restoring moment at the base of tactile hairs is roughly up to 4 powers of 10 larger than in trichobothria. As a consequence, the hair shaft of tactile hair is bent by the stimulating force [56]. Therefore, for applied research on biosensor types, more information about the size and structure of the particular types of mechanosensilla is needed.

In the present study of mechanosensillum in *N. rugosus*, the coupling and transduction mechanisms are deduced from Figure 8DVI, indicating that the dendritic sheath terminal was connected to the left side of the hair base, while another part of the sheath was linked to the socket through the fibrous material socket septum to prevent the dendrite sheath from moving. If the hair moves leftward due to the airflow or any mechanical disturbances, the hair’s base moves to the right side, and the hair base at connection may readily squeeze the dendrite sheath. Since the socket septum supports the dendrite sheath from not moving, the hair base pushing the dendrite sheath produces action potentials from the tubular body and its microtubules. When the hair returns to its resting position, the hair base departs from the dendritic sheath, and the tubular body is unaffected, with no generation of action potential. According to the authors [57] here, the mechanical stimuli are converted into action potentials, so this tubular body inside the dendrite sheath connected to the hair base is considered a transduction site. Microtubules work in a suspension system. As we know, due to the mechanical excitation of hair, the coupling mechanism of the hair base and the dendrite sheath transforms the stimuli into displacements of the dendrite sheath, by which the electrical potential develops in the microtubule core. The developed electrical potential depends on the value of the exciting force and is different at each microtube in its core, leading to the feedback analysis. This analysis can be investigated by formulating the numerical model of the mechanosensors using multiphysics analysis software.

## 5. Conclusions

The findings are preliminary to the external morphology of the trichoid sensilla, which exhibits different lengths and distributions on pedicel and flagellomere antennal segments of *N. rugosus* using SEM.

This study presents characteristics of the five main types of antennal sensilla, namely trichoideum, chaeticum, campaniformium, coeloconicum, and basiconicum, and essentially focuses on the ultrastructure of the receptor in the base of the mechanosensillum (trochoidal hair) positioned inclinedly to the cuticular surface and the reconstruction of a 3D model using TEM data. The study’s novelty shows a microtubule quantity of ninety in number at the terminal tubular body, its position at the end of the dendritic sheath, and the path of the dendrite traversed from the epidermal layer by the cuticular layer to the base of the trichoid hair. Moreover, this study compares the sensillum length to the tubular body diameter and the number of microtubules and discusses the parameters of this mechanosensillum in contrast to other insect species (*N. lugens*). These data show the dependencies between external and internal structures of the mechanoreceptors in different insect species. This study compares the sensillum length to the tubular body diameter and the number of microtubules. The length of the sensillum in *N*. *rugosus* is about 25 µm long, and it has a long socket axis of about 11.3 µm, which is longer than the sensillum of the *N. lugens*, whose length is 11.8 µm, with a socket axis of about 4.8 µm. In *N. rugosus,* the diameter of the tubular body in the trichoid sensillum is significantly larger, which is 150 nm compared to 50 nm for *N. lugens*, and nearly 90 microtubules are found from the terminal of the tubular body in *N. rugosus*, whereas 42 microtubules are found in *N. lugens*. The detailed knowledge of the structure of a specific mechanosensillum impacts the selection and preparation of future models of operation of such mechano-sensors for other fields of science and practical applications in various areas.

The study shows the subcellular/cellular organisation of the trichoid sensillum hair of *Nabis* and the mechanics of the sensillum located obliquely on the surface of the antennae. The sensillum base is embedded in the socket and jointed via the membrane, which is less developed from the dorsal side of the hair than the ventral side, similarly to an area of the suspension fibres. The dendrite passes through the well-developed tormogen cell, and socket septum and is connected more laterally at the hair base. The 3D model obtained will be numerically evaluated in response to external stimulation over the hair, and analysis will be performed on the dendrite sheath’s mechanical deformation, which generates sensing signals. In developing a highly accurate and sensitive artificial, bioinspired sensors model, this study of a reconstructed 3D model and its biological sensing operations is helpful.

## Data Availability

The data presented in this study are available on request from the corresponding author.
